# Genetic variants conferring susceptibility to Alzheimer’s disease in the general population; do they also predispose to dementia in Down’s syndrome

**DOI:** 10.1186/1756-0500-7-42

**Published:** 2014-01-17

**Authors:** Ashok Patel, Simon D Rees, M Ann Kelly, Stephen C Bain, Anthony H Barnett, Anisha Prasher, Humaria Arshad, Vee P Prasher

**Affiliations:** 1 Department of Biomolecular and Sport Sciences, Faculty of Health and Life Sciences, Coventry University, Coventry, UK; 2 College of Medical and Dental Sciences, University of Birmingham, Birmingham, UK; 3 Institute of Life Science, Swansea University, Swansea, UK; 4 Diabetes Centre, Heart of England NHS foundation Trust, Birmingham, UK; 5 Nottingham University, Nottingham, UK; 6 South Birmingham Primary Care Trust, Birmingham, UK; 7 Liverpool John Moore University, Liverpool, UK

**Keywords:** Down syndrome, Dementia, Alzheimer’s disease, SNPs

## Abstract

**Background:**

Down’s syndrome (DS) is caused by either complete or partial triplication of chromosome 21, affecting approximately 1/1000 live births, and it is widely accepted that individuals with DS are more likely to develop dementia of Alzheimer’s disease (DAD) compared with the general population. Recent collaborative genome-wide association studies of large case control data sets of individuals with and without Alzhemier’s disease (AD) have revealed new risk variants for dementia, as well as confirming previously identified risk variants. In this study, nine AD-derived SNPs, near or within the *CR1* (rs3818361), *BIN1* (rs744373), *CD2AP* (rs9349407), *EPHA1* (rs11767557), *CLU* (rs1532278), *MS4A6A/4A* (rs610932), *PICALM* (rs561655), *ABCA7* (rs3764650) and *CD33* (rs3865444) genes were genotyped in 295 individuals with DS.

**Results:**

There were no significant associations between these nine GWAS-derived SNPs and DAD in British Caucasian individuals with DS. Interestingly the *CR1* rs3818361 variant appeared to be associated with mortality in our cohort, particularly in the subjects without dementia. To our knowledge, this is the first time that this variant has been implicated as a determinant of mortality and the finding warrants further investigation in other cohorts with DS.

**Conclusions:**

This study shows negative associations of nine AD-derived SNPs with DAD in DS. This may be due to the modest size of our cohort, which may indicate that our study is insufficiently powered to pick up such associations. We cannot conclusively exclude a role for these SNPs in DAD in DS. Clearly, efforts to investigate genetic variants with small effects on disease risk require a much larger cohort of individuals with DS. In fact, we hypothesize that a sample size of 4465 individuals with DS would be needed to determine the role in DAD in DS of the nine AD-derived SNPs investigated in this study. We therefore recommend that all national and international clinics with access to individuals with DS should contribute DNA samples to form DS consortia.

## Background

Down’s syndrome (DS) is caused typically by a complete, or occasionally partial, triplication of chromosome 21 and has an incidence of approximately 1/1000 live births. The phenotype is complex and variable, primarily characterised by cognitive and language dysfunction coupled with sensory and neuromotor deficits and a neuropathology resulting in decreased brain size and weight [1]. DS is also characterised by congenital heart and bowel problems [2] and increased mortality rates both in early and later stages of life [3].

Evidence indicates that the dementia in DS is in fact Alzheimer’s disease (AD) [4,5]. It is widely accepted that individuals with DS are much more likely to develop dementia of AD (DAD) than the general population, especially over the age of 35 years, [6,7]. Virtually all individuals with DS are likely to develop the neuropathological changes characteristic of AD by the age of 40 years; these include deposits of extracellular beta-amyloid in neuritic plaques and intracellular neurofibrillary tangles [8]. However, despite the nearly universal occurrence of AD pathology by this age, it is clear that not all of these individuals develop clinical psychopathology of AD. A number of studies have shown that there is wide variation in the age of onset of dementia, from 38 to 70 years, with an average age at onset between 50 and 55 years [9,10]. This variation may be explained by a number of factors, including methodological difficulties in diagnosis of DAD in this population [11], triplication of the beta-amyloid precursor protein (APP) gene [12], *APOE* genotypes [6,13], gender [14] and a number of unknown genetic variants.

The neuropathological manifestations of DAD in DS have been attributed to triplication and over-expression of the APP gene located on chromosome 21 [12]. However, factors other than APP triplication must be responsible for the individual differences in susceptibility to the formation of fibrillised plaques and for the wide range in age at onset of dementia [6]. In the general population the gene coding for apolipoprotein E (*APOE*) on chromosome 19 has been shown to modulate the risk of AD in some studies. The ϵ4 allele of APOE is associated with earlier age at onset and increased risk of AD [15-19] whilst the APOE ϵ2 allele may reduce the risk of dementia in heterozygous carriers [20,21].

A number of studies have shown an association between the APOE ϵ4 allele and an increased risk of DAD in individuals with DS [6,13,22,23], but other studies have shown conflicting results [24-26]. There is evidence that APOE ϵ2 is associated with increased longevity in DS [27,28] and a potential protective effect on DAD [28-30], but these effects have not been confirmed by all studies [13,22,23]. Similarly, the influence of the APOE ϵ4 allele on early mortality in individuals with DS [13,31] is subject to dispute [32].

A number of genes besides APOE have been implicated in susceptibility to DAD in individuals with DS, including TNF-α [24], BACE 1 [33], PSEN [34], SORL1 [35], ALDH18A1 and RUNX1 [36].

Recent collaborative genome-wide association studies of large case control data sets of individuals with AD have revealed new risk variants for dementia, as well as confirming previously identified risk variants [36,37]. Our present understanding of the genetic architecture of AD suggests that at least ten loci contribute to disease risk; *APOE*, *CR1, CLU*, *PICALM*, *BIN1*, *EPHA1*, *MS4A*, *CD33*, *CD2AP* and *ABCA7*. As described above, the contribution of *APOE* to predisposition to dementia in DS has been widely studied [38], but the influence of the other nine risk variants on the development of DAD has yet to be investigated. The aim of our study, therefore, was to characterise the contribution of these AD susceptibility loci in the general population to the risk of DAD in a cohort of individuals with DS, to determine whether the two diseases have a common genetic basis.

## Methods

### Participants

Three hundred and four adults with DS (16 years and above) of white Caucasian ancestry, known to the local clinical services and voluntary organizations, were recruited into the study. Consent or assent was obtained as was appropriate. All participants resided in the West Midlands, United Kingdom. Ethical Committee approval was obtained from the local authority with approval from the NHS Trust.

### Procedures

A prospective cohort design was used, with a range of two to 14 sequential assessments over the course of the follow up. Approximately 80% of participants had cytogenetic tests. Of these, 222 had trisomy 21, 12 had trisomy 21 translocation, 7 had trisomy 21 mosaicism, 1 had partial trisomy 21 and 1 had trisomy 21 and 48XXY. Some individuals did not consent to give blood for cytogenetic tests, or blood could not be obtained from them. Baseline assessment included a standard full psychiatric history and mental state examination. Mental disorders were diagnosed using the ICD-10 Symptom Checklist for Mental Disorders [39] according to ICD-10 research criteria [40]. An ascertainment of severity of dementia according to ICD-10 criteria [41] was made along with a physical examination (including an assessment of hearing and vision). There was also a comprehensive review of medical records, haematological, biochemical, and thyroid screening and a comprehensive review of all prescribed medications. Participants diagnosed with mental or physical disorders were treated appropriately and then followed up. Dementia in Alzheimer’s disease was determined according to ICD-10 research criteria [40].

### SNP selection and genotyping

Nine SNPs that had previously been robustly associated with late-onset Alzheimer’s disease (*p* < 5 × 10^-8^) were genotyped in 295 subjects with Down’s syndrome. These SNPs were in or near the *CR1* (rs3818361), *BIN1* (rs744373), *CD2AP* (rs9349407), *EPHA1* (rs11767557), *CLU* (rs1532278), *MS4A6A/4A* (rs610932), *PICALM* (rs561655), *ABCA7* (rs3764650) and *CD33* (rs3865444) genes [37]. Genotyping was performed by KBioscience (Hertfordshire, UK) using the KASP™ genotyping method (http://www.lcggenomics.com/genotyping). The DNA samples of 268 subjects, both with (N = 109) and without (N = 159) dementia, demonstrated genotyping success rates greater than 75% over the 9 SNPs and were included in the main analyses. One hundred and twenty-five samples (47%) were genotyped in duplicate for all SNPs. Genotypes were called independently and an error rate of zero was observed.

### Statistical analyses

Cox proportional hazard regression was used to assess the effect of each SNP on the risk of dementia and mortality, including sex and level of intellectual disability as covariates. Age of onset of dementia or age at last assessment, or age at death or age at last assessment, were the time-to-event variables. As the minimum age at onset of dementia in the study was 38 years, only those non-demented subjects who were = > 38 years of age at last assessment were included in the risk of DAD in DS analyses (N_total_ = 225). The earliest age of death was 36 years, therefore only those surviving subjects who were = > 36 years of age at last assessment were included in the mortality risk analysis (N_total_ = 233). Mortality risk was estimated both in the entire cohort and within the subset of individuals who remained non-demented throughout the period of the study. SNPs were analysed using an additive model, coding each genotype as 0, 1 or 2 Alzheimer’s disease risk alleles. The single Hazard Ratio (HR) given for each SNP therefore represents a per risk allele effect.

For the risk of DAD in DS analyses (N_event_ = 109), given minor allele frequencies of between 5–50%, this study had 80% power to detect HRs in the region of 3.43–1.71. Respective estimates for the mortality analyses (N_event_ = 80), given the same allele frequencies, were 4.21–1.87. Given the modest effect sizes of the studied SNPs on the risk of Alzheimer’s disease (reported odds ratios of 1.1-1.23[36,37]), it was unlikely that adequate statistical power could be achieved for the analysis of individual SNPs in DAD using the current study’s modest sample size. We therefore created a genetic risk score (GRS), calculated as the total number of Alzheimer’s disease risk alleles within each individual, using those subjects with a 100% genotyping success rate (N = 243). The GRS was used to investigate whether genetic susceptibility for Alzheimer’s disease in the general population overlapped with that for DAD in DS. All DAD in DS analyses were adjusted for *APOE* using SNP rs429358 as a proxy. Throughout this manuscript associations are referred to as nominally significant (*p* < 0.05) or study-wide significant (corrected for the 9 independent SNPs; p < 5.5 × 10^-3^). All analyses were conducted using R (http://www.R-project.org) or STATA IC V10.1 (Stata Corporation, College Station, TX, USA).

## Results

### DAD in DS

One SNP was nominally associated with the development of DAD in DS; the *PICALM* rs561655 variant appeared to reduce the risk of DAD (HR = 0.72, p = 0.02; Table 1). In contrast, six of the nine variants had hazard ratios greater than 1, although none of these was significantly associated with the development of dementia (Table 1). The combined GRS was not associated with DAD in DS (HR = 0.98, *p* = 0.78; Table 1).

**Table 1:** Association between SNPs and DAD in DS

**Nearest gene**	**SNP**	**Risk allele**	**Hazard ratio**	**SE**	***p*-value**
***CR1***	rs3818361	A	1.10	0.17	0.59
***BIN1***	rs744373	G	1.16	0.16	0.36
***CD2AP***	rs9349407	C	0.99	0.15	0.93
***EPHA1***	rs11767557	T	1.03	0.16	0.86
***CLU***	rs1532278	C	1.05	0.13	0.69
***MS4A6A***	rs610932	G	1.03	0.15	0.83
***PICALM***	rs561655	A	0.67	0.14	5.89 × 10^-3^
***ABCA7***	rs3764650	G	1.09	0.29	0.76
***CD33***	rs3865444	C	0.83	0.15	0.22
**Genetic risk score**	-	–	0.97	0.06	0.66

Covariates included in the analyses were sex, intellectual disability and *APOE* genotype.

### Mortality

In the overall cohort, the *PICALM* rs561655 variant was nominally associated with a reduced risk of mortality (HR = 0.71, *p* = 0.048) whilst the *CR1* rs3818361 variant increased the risk of early mortality (HR = 1.65, *p* = 5.36 × 10^-3^) (Table 2); this latter association achieved study-wide significance. In the non-demented group, the effect of the *CR1* variant was more pronounced (HR = 2.33, *p* = 4.08 × 10^-3^), whilst that of the *PICALM* SNP disappeared. Variants in or near *CD33* and *CD2AP* also demonstrated weak but nominally significant associations with mortality (Table 2).

**Table 2:** Association between genetic variants and mortality

		**Risk allele**	All subjects (N = 233)*			Subjects with Dementia (N = 109)**			Subjects without dementia (N = 124)**		
**Nearest gene**	**SNP**	A	**Hazard ratio**	**SE**	***p*-value**	**Hazard ratio**	**SE**	***p*-value**	**Hazard ratio**	**SE**	***p*-value**
***CR1***	rs3818361	G	1.65	0.18	5.36 × 10^-3^	2.33	0.29	4.08 × 10^-3^	1.35	0.23	0.19
***BIN1***	rs744373	C	1.05	0.20	0.82	1.34	0.44	0.51	0.94	0.25	0.81
***CD2AP***	rs9349407	T	1.10	0.18	0.61	1.92	0.31	0.04	0.82	0.23	0.40
***EPHA1***	rs11767557	C	1.23	0.19	0.29	0.76	0.35	0.44	1.47	0.24	0.10
***CLU***	rs1532278	G	1.17	0.16	0.33	0.89	0.32	0.73	1.27	0.20	0.23
***MS4A6A***	rs610932	A	0.72	0.18	0.07	1.02	0.34	0.95	0.64	0.21	0.03
***PICALM***	rs561655	G	0.71	0.17	0.05	0.94	0.36	0.86	0.64	0.21	0.04
***ABCA7***	rs3764650	C	1.08	0.33	0.81	1.27	0.54	0.65	0.74	0.46	0.52
***CD33***	rs3865444	–	0.73	0.18	0.08	0.53	0.33	0.05	0.91	0.21	0.67
**Genetic risk score**	-		1.70	0.25	0.03	3.13	0.52	0.03	1.54	0.28	0.13

*Covariates included in the analyses were sex, intellectual disability and dementia; **Covariates included in the analyses were sex and intellectual disability.

The GRS was not significantly associated with mortality in either the overall cohort or the non-demented subset (Table 2).

## Discussion

To our knowledge, this is first study to investigate the effects of nine of the GWAS-derived susceptibility variants for Alzheimer’s disease in the general population on the development of DAD in DS. The *CR1* (rs3818361), *BIN1* (rs744373), *CD2AP* (rs9349407), *EPHA1* (rs11767557), *CLU* (rs1532278), *MS4A6A/4A* (rs610932), *ABCA7* (rs3764650) and *CD33* (rs3865444) SNPs showed no significant association with dementia in our cohort of individuals with DS, either singly or as part of a genetic risk score. A nominally significant association was observed with the *PICALM* rs561655 variant, but the direction of effect was opposite to that expected from earlier studies of Alzheimer’s disease.

Interestingly the *CR1* rs3818361 variant appeared to be associated with premature mortality in our cohort, particularly in the subjects without dementia. To our knowledge, this is the first time that this variant has been implicated as a determinant of early mortality and the finding warrants further investigation in other cohorts with DS.

There is considerable evidence that there is a great deal of similarity between AD and dementia in DS [5] and it would be reasonable to expect a similar genetic predisposition in both conditions. A number of published studies that have investigated the genetic predisposition to DAD in DS and the sizes of cohorts with DS in these studies have ranged from 52 to 425 [22,30]. These studies, like the current investigation, were underpowered to detect effect sizes of the magnitude reported for recent Alzheimer’s disease susceptibility variants. Replication studies have been undertaken to detect genetic variants associated with AD using relatively large numbers of individuals with AD and matched controls. When case control cohort sizes were examined in three such robust studies, they have ranged from 1829 to 3287 individuals with AD in the general population, and 2576 to 4396 matched controls [42-44] Given a hypothetical genetic variant associated with DAD in DS with a HR of 1.15 and a minor allele frequency of 0.25 (not unreasonable estimates based upon the large Alzheimer’s disease GWAS previously mentioned) and a ‘failure rate’ of 0.48 (the proportion of subjects who developed dementia in the present study), to have 80% power at an alpha of 0.05 we would need a sample size of 4465 subjects with DS. Due to this lack of power we cannot conclusively exclude a role for these SNPs in DAD in DS, despite the lack of association demonstrated in this study. It is also possible that the lack of significant associations that we observed are due to the fact that only one variant per locus was investigated. The variants identified to be associated with Alzheimers’s disease in recent GWAS are not aetiological variants [37], the hypothesis being that they are proxies in linkage disequilibrium with variants that exert a direct effect. It is therefore possible that by restricting our coverage of the loci in question, we may have missed associations with other genetic variants, due to differences in LD patterns. Investigation of a greater number of variants on these susceptibility loci is needed in future studies.

Clearly efforts to investigate genetic variants with small effects on disease risk require a much larger cohort of individuals with DS. A UK national database and DNA resource for DS would be an invaluable resource for researchers to elucidate risk alleles for DAD in DS. We therefore recommend that all national and international clinics with access to individuals with DS should contribute DNA samples to form DS consortia. These resources would facilitate well-powered genome-wide genetic studies to elucidate the risk variants that contribute to DAD in DS and investigate associations with risk variants implicated in other forms of dementia.

## Conclusions

Nine SNPs, previously shown to contribute to the predisposition to AD, showed no association with DAD in DS in this study. However, we cannot exclude a role for these SNPs in DAD in DS, as our study is insufficiently powered to pick up genetic variants with small effects on disease.

## Competing interests

The authors declare that they have no competing interests.

## Author’s contributions

VP and HA collected blood from individuals with DS and undertook mental health assessments, SDR did the statistical analysis and contributed to drafting of paper, A Patel, SDR and VP contributed to the design of the study, A Patel drafted the paper, MAK contributed to drafting of the paper, SCB initiated the original idea to examine the DS cohort for genetic risk for dementia and along with AHB contributed to the drafting of the paper. AP undertook data collection. All authors read and approved the final manuscript.
